# An overview of the effect of bioaerosol size in coronavirus disease 2019 transmission

**DOI:** 10.1002/hpm.3095

**Published:** 2020-12-08

**Authors:** Marcelo I. Guzman

**Affiliations:** ^1^ Department of Chemistry University of Kentucky Lexington Kentucky USA

**Keywords:** aerodynamic size, bioaerosol, COVID‐19, infection, SARS‐CoV‐2, social distancing, virus

## Abstract

The fast spread of coronavirus disease 2019 (COVID‐19) constitutes a worldwide challenge to the public health, educational and trade systems, affecting the overall well‐being of human societies. The high transmission and mortality rates of this virus, and the unavailability of a vaccine or treatment, resulted in the decision of multiple governments to enact measures of social distancing. Such measures can reduce the exposure to bioaerosols, which can result in pathogen deposition in the respiratory tract of the host causing disease and an immunological response. Thus, it is important to consider the validity of the proposal for keeping a distance of at least 2 m from other persons to avoid the spread of COVID‐19. This work reviews the effect of aerodynamic diameter (size) of particles carrying RNA copies of severe acute respiratory syndrome coronavirus 2 (SARS‐CoV‐2). A SARS‐CoV‐2 carrier person talking, sneezing or coughing at distance of 2 m can still provide a pathogenic bioaerosol load with submicron particles that remain viable in air for up to 3 h for exposure of healthy persons near and far from the source in a stagnant environment. The deposited bioaerosol creates contaminated surfaces, which if touched can act as a path to introduce the pathogen by mouth, nose or eyes and cause disease.

## 1 INTRODUCTION TO COVID‐19 PANDEMIC

The recent global spreading of a novel coronavirus disease 2019 (COVID‐19), caused by the severe acute respiratory syndrome coronavirus 2 (SARS‐CoV‐2), constitutes an unprecedented challenge in recent history to the international public health, educational and trade systems, affecting the overall well‐being of human societies.^1^ By 26 May 2020, the pandemic of COVID‐19, as labelled by the World Health Organization (WHO),^2^ has caused more than 5,555,691 cases of COVID‐19 in at least 188 countries, resulting in more than 348,541 deaths and more than 2,271,268 recoveries.^3^ The high transmission and mortality rates of this virus, and the unavailability of a vaccine or effective treatment, resulted in the decision of multiple governments to force social distancing and related measures (e.g., telecommuting and homeschooling) as friendly alternatives to the enforcement of strict quarantine of affected areas. The idea of establishing a physical distancing is to slow down and eventually stop the spread of the pathogen by reducing the probability of contact between persons carrying SARS‐CoV‐2 and others who are not infected.^4^

Recent epidemiological information indicates an incubation period of COVID‐19 of 1–14 days, with active transmission during the latency period, especially to persons with underlying diseases and elders.^5^ Among the different symptoms exhibited by COVID‐19, patients are included fever, headaches, sore throat, diarrhoea, vomiting, loss of taste and smell, red eyes, shortness of breath, fatigue, bright red to purple toes, chest pain and dry cough; with extreme conditions extending quickly to acute respiratory distress syndrome, respiratory failure, multiple organ failure and even death.^6^, ^7^, ^8^, ^9^, ^10^ Despite the loss of benefits from human interactions, social distancing^4^ is expected to be an effective mechanism to prevent the COVID‐19 infection via bioaerosol contact (e.g., from talking, coughing or sneezing),^11^ ^,^ ^12^ and by indirect physical contact by touching a contaminated surface (e.g., a fomite). It must be noted here that the term bioaerosol is used with the medical meaning of a tiny, airborne particle that is composed of or derived from biological matter,^13^ which can spread infectious diseases by carrying viruses (e.g., the influenza A H1N1 virus,^14^ and SARS virus).^15^ This airborne transmission (meaning transmission by particles of aerodynamic diameter <10 μm ^16^) of COVID‐19 could, in theory, also originate from particulates emitted during vomiting and toilet flushing (e.g., toilet water aerosolization) following fecal excretion.^17^

The focus of the information below is on the bioaerosol emission from persons carrying SARS‐CoV‐2, who aerosolize particles and on the resuspended dust with the pathogen,^18^ both containing aggregates of the virus,^11^ which are categorized by their aerodynamic diameter. The source mechanism of bioaerosols emitted by humans constrains the particle size distribution. Normal breathing creates particles in the <0.8–2.0 µm range.^19^ While speaking, two size distributions have been observed, 16–125 µm^20^ ^,^ ^21^ and <0.8–7.0 µm^19^ with a mean of 1.0 µm for shouting.^22^ Similarly, coughing also displays a dual range of 0.6–16 µm^19^ ^,^ ^20^ ^,^ ^23^ ^,^ ^24^ and 40–125 µm.^21^ ^,^ ^25^ Sneezing largely contributes particles in the 7–125 µm range.^25^ ^,^ ^26^ Even though humans can only inhale particles <100 μm, it should be considered that the initial larger particles can undergo rapid evaporation depending on the environmental relative humidity.^27^, ^28^, ^29^ This work is focused on the factors governing particle size, deposition site, clearance and inhalational infection of COVID‐19. The final inhaled particle size depends among other factors on the solid organic content of the original particle including the virus and the distance of an individual to the bioaerosol source. Other factors that impact on air mass movement (e.g., ventilation) may offset the terminal velocity of particles in still air.^27^ ^,^ ^29^ This work is focused on discussing the generation and transport of bioaerosols with pathogenic SARS‐CoV‐2 by talking, breathing, sneezing and coughing as governed exclusively by particle size (in the range from <1 to >100 μm). The work connects the particle size distribution to deposition in the respiratory system causing infection.

## 2 SAMPLING METHODS AND CHARACTERIZATION OF BIOAEROSOLS WITH SARS‐CoV‐2 RNA

The complete genome of a strain of SARS‐CoV‐2 totaling 29.9 kb was characterized from a pneumonia patient with COVID‐19 in Wuhan.^30^ SARS‐CoV‐2 (50–200 nm virion diameter)^31^ can be transmitted through human respiratory bioaerosols and direct contact with infected persons. The aerosol transmission of SARS‐CoV‐2 has been recently reported from the analysis of 35 aerosol samples collected from patients and medical staff areas in Wuhan and Fangcang hospitals in China.^18^ Three different types of aerosols were studied during this originating COVID‐19 outbreak, (1) total suspended particles, (2) size segregated (>2.5 μm, 1.0–2.5 μm, 0.50–1.0 μm and 0.25–0.50 μm, and 0–0.25 μm) and (3) deposition aerosol.^18^ For the size‐segregated aerosol samples,^18^ a miniature cascade impactor (Sioutas impactor, SKC Inc) consisting of four impaction stages and an after‐filter was employed in combination with a pump operating at flow of 9 L min^−1^.^32^ ^,^ ^33^ The total pressure drop created across the sampler (2.7 kPa), allows the impactor to efficiently separate and simultaneously collect (coarse, fine and ultrafine) airborne particles in the five size ranges listed.^32^ ^,^ ^33^ The selective determination of the concentration of aerosol with viral SARS‐CoV‐2 RNA was enabled by a droplet digital polymerase chain reaction (ddPCR) method.^18^ Related studies have been proposed in the future using enhanced virus culture techniques and alternative protocols for the collection of size fractionated particles for the detection of SARS‐CoV‐2 in air samples.^34^

**FIGURE 1:**
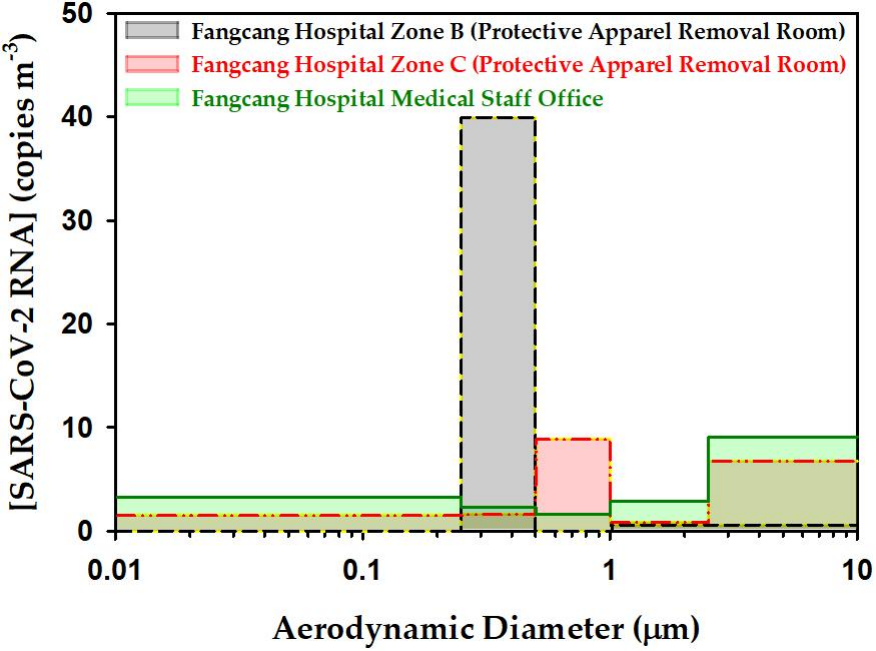
Concentration of airborne severe acute respiratory syndrome coronavirus 2 (SARS‐CoV‐2) RNA in aerodynamic size‐segregated aerosol samples at three different locations of Fangcang Hospital as reported in ref.^18^

Figure 1 displays the relatively high concentration of SARS‐CoV‐2 RNA in two protective apparel removal rooms and a medical staff office of the Fangcang Hospital. Each zone of the Fangcang Hospital hosted >200 patients at the peak of the COVID‐19 outbreak. Based on the information of this hospital setting, there are two size ranges with high concentration of SARS‐CoV‐2 aerosols (Figure 1), one dominated by submicron aerodynamic diameter particles (0.25–1.0 µm), and another by particulates with diameter >2.5 µm.^18^ The maximum concentrations in the so‐called apparel removal zones (B and C) were ∼40 and 9 copies of SARS‐CoV‐2 RNA per cubic metre in the 0.25–0.5 µm, and 0.5–1.0 µm intervals, respectively.^18^ Instead, maxima concentrations of 7 and 9 SARS‐CoV‐2 RNA copies m^−3^ for supermicron particles were identified in the apparel removal zone C and the medical staff office.^18^ Submicron and supermicron aerosol carrying the pathogen can coexist due to the variable generation pathways. The supermicron aerosols in the apparent bimodal size distribution of SARS‐CoV‐2 particulates (from apparel removal zone C) is likely associated with the resuspension of deposited pathogenic dust that generates a secondary aerosol. Resuspension of pathogenic particles^35^ from the protective equipment and shoes can occur during movements (e.g., walking, sitting, removing clothing, etc.), and when handling and folding written records of patients or reordering their assigned space and belongings.

Because the aerosolized copies of the virus deposited on the protective apparel of medical staff during their long working hours can be resuspended during movements, a prior sanitation step before removal is needed.^18^ The deposition of the pathogen resulting from particulates emitted during respiration, coughing and sneezing may also cause infection of people touching the contaminated surfaces. For example, related measurements of aerosol deposition rates for samples collected ≥2 m far from patients beds inside an intense care unit at the Renmin Hospital were up to 31 and 113 SARS‐CoV‐2 RNA copies m^−2^ h^−1^.^18^ The infectious dose of SARS‐CoV‐2 particles needed to cause COVID‐19 is not known yet but should be expected to be relatively low (100–1000 particles) as the disease spreads rapidly.^36^ The high basic reproduction number (*R*_*0*_), indicating the average number of people one infected person has been passing the SARS‐CoV‐2 virus to others, is in the range 2 ≤ *R*_*0*_ ≤ 2.5.

## 3 AERODYNAMIC SIZE EFFECT ON DEPOSITION AND CLEARANCE ON THE RESPIRATORY SYSTEM

**FIGURE 2:**
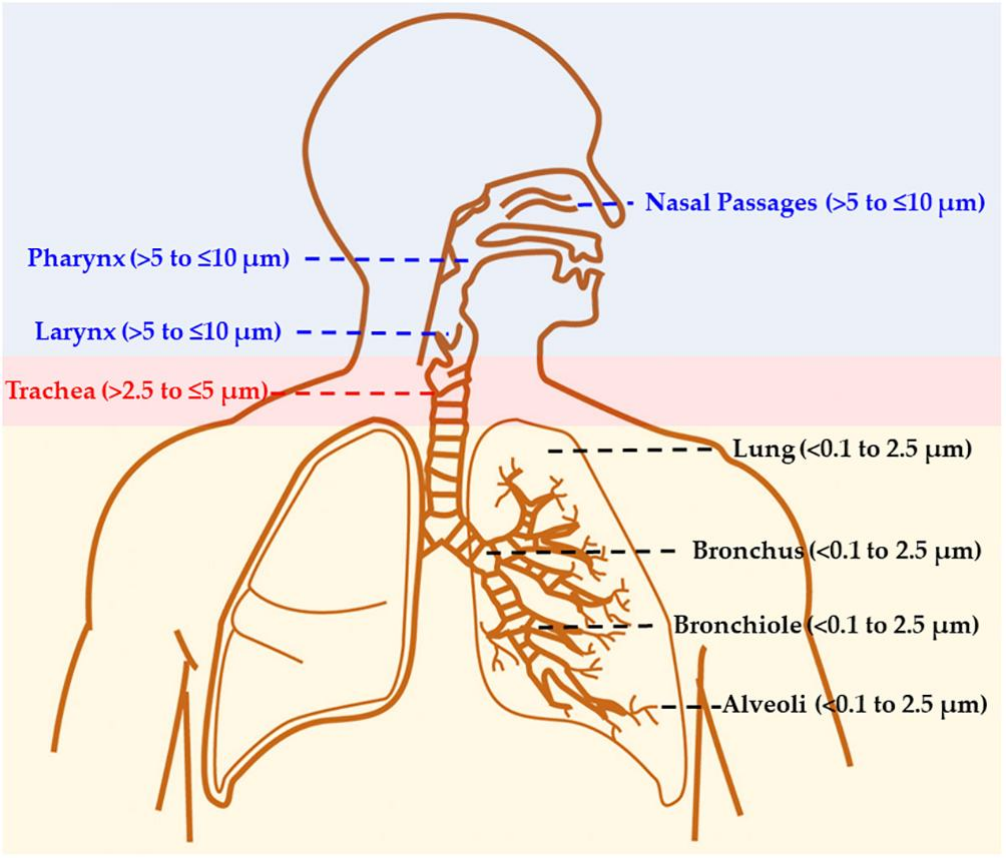
Schematic representation of the respiratory system with the corresponding aerodynamic diameter size dependent penetration of particles

The aerodynamic diameters reported in Figure 1 are key to determine where the inhaled particles with the pathogen are deposited in the respiratory tract of an exposed person. Various deposition mechanisms can exist, including inertial impaction, gravitational settling, Brownian motion, turbulent deposition, interception and electrostatic attraction.^37^ The smallest particles (<1–2.5 μm) can diffuse directly deep into the lung tissue, where they get deposited on the alveoli by diffusion, sedimentation and electrostatic attraction. Instead, inertial impaction in the upper airways determines that the largest particles (>8 μm) are size dependently deposited from the nasal passage to the bronchioles. Multiple factors (e.g., age, weight, sex, physical activity level and disease state) impact respiration and deposition profiles.^38^ Larger particles can be inhaled into the respiratory tract under exertion breathing because the oral cavity is larger and results in bypassing of the nasal cavity filtration mechanism.^38^ Figure 2 is used to briefly illustrate known information of the respiratory system, which is needed for the discussion of possible deposition sites affecting the clearance of tissues and infection rates for inhaled SARS‐CoV‐2 pathogen. Descriptive computational models developed to predict the deposition of aerosol particles inside the lung (with a reasonable accuracy relative to experimental data) have been discussed in a recent review.^39^ Assuming good hygiene protocols are put in place to avoid spreading of COVID‐19 by direct contact, the major mechanism of infection involves bioaerosols and respiratory secretions.^40^ Particulates with SARS‐CoV‐2 RNA in the <1–10 µm range of aerodynamic diameter can penetrate into the respiratory tract through the nose and/or mouth (Figure 2), from where it can be further disseminated. Effective filtering in the nose prevents large supermicron particles >5 µm to penetrate further in than the nasal, pharyngeal and laryngeal regions. The virus in supermicron particles deposited in the nasopharyngeal region can pass through the mucous membranes^41^ to replicate and continue spreading to the lungs. Particles in the 2.5–5 µm range are deposited in the tracheas, while fine (≤2.5 µm) and ultrafine particles (≤0.1 µm), due to their small size, reach deep into the lungs, to be deposited in the alveolar ducts and sacs. Fine and ultrafine particles may cause direct COVID‐19 transmission, as suggested by epidemiological data for three family clusters exposed to SARS‐CoV‐2 in a restaurant, which could not be explained by >5 µm particles alone, but required invoking virus‐laden aerosols (<5 μm).^42^

Most of the particles with SARS‐CoV‐2 RNA in the ranges 0.25–0.5 µm and 0.5–1 µm in both protective apparel removal rooms (Figure 1) can be directly inhaled to the lungs as well as travel long distances in air. The transport of these smaller viral particles (meters and tens of meters from their source) by air currents is more favorable than sudden gravitational deposition and varies largely with evaporative loss and condensation gain of liquid content of particles.^43^ Instead, abundant particles from 2.5 to 10 µm in the medical staff office and protective apparel removal zone C are only deposited on (and start the infection in) the nasal, pharyngeal and laryngeal regions and the trachea.

As for other respiratory diseases, COVID‐19 transmission from exposure of exhaled particles should include both droplet‐borne (large droplet, >5 µm) and short‐range (droplet nuclei, <5 µm) airborne routes, which are affected by distance, humidity, ventilation and breathing mode.^44^ For example, related studies indicated that coughing particles <4 µm contained 65% of exhaled influenza virus,^45^ suggesting the airborne route for COVID‐19 transmission could be dominant for exhaled particles.^44^ Aerosol transmission and inactivation of related infectious influenza A viruses are known to depend on humidity levels.^46^ The shrinking of respiratory droplet size when the humidity drops from 90% to 10% in 10 min was used to explain the accompanying a 2.4‐times increase in virus concentration; suggesting the importance of keeping a high humidity indoors to prevent the spreading of diseases^46^ such as COVID‐19. Exhaled breath of symptomatic patients aerosolizes influenza virus without the need for coughing and sneezing, causing compartmentalized and independent infection in the upper and lower airway.^47^ Particularly, exhaled particles <5 µm in size contributing to infecting directly the lungs, triple the influenza RNA concentration (3.8 × 10^4^ copies in 30 min) measured for >5 µm particles.^47^

It is believed that angiotensin‐converting enzyme 2 (ACE2), an abundant protein on epithelial cells of the lung alveolar surface, kidney and heart cells, as well as of enterocytes in the small intestine,^48^ plays a major role during infection. The S protein on the surface of SARS‐CoV‐2 is recognized by the target cell and binds to ACE2 for the virus to then penetrate, replicate until it kills the cell, and cause infection.^49^ The detailed infection mechanism, the strength of the interaction needed to cause COVID‐19 transmission, and the pathological processes damaging organs are the topic of current studies.^49^

The speed of clearance of deposited supermicron particles depends on the exact location in the nasopharynx and in the person's health state. Fundamental studies have shown that the typical clearance from the ciliated anterior region of particles with other pathogens occurs in the range from 1.3 to 12.6 mm min^−1^, which is considerably faster than for the non‐ciliated posterior region.^50^ Similarly, clearance from the tracheal and bronchial mucociliary are in the range from 0.8 to 12.4 mm min^−1^.^51^ ^,^ ^52^ The presence of mucus on the surface of the nasal and tracheobronchial regions typically serve to capture deposited particulates, which are then removed to the gastrointestinal tract via the cumulative action of the cilia. The viscoelasticity, wettability and adhesiveness of the mucus depend on its variable composition of glycoproteins (mucins), proteins, proteoglycans and lipids. Therefore, the concentration of these components controls the size of particles emitted by coughing or sneezing.^53^, ^54^, ^55^ The specific structure of the oligosaccharide chains present on these respiratory mucins and proteoglycans can play a major role and provide a pathway for pathogen interaction and clearance.^56^

It is well known that SARS‐CoV‐1, the predecessor for SARS‐CoV‐2 virus, spread by aerosol particles in air,^15^ ^,^ ^57^ ^,^ ^58^ and that airborne transmission was key in indoor cases. Based on the stability of SARS‐CoV‐2 in aerosols generated in the lab using a 3‐jet Collison nebulizer (<5 μm at 65% relative humidity at 22 (±1) °C),^59^ the majority of the aerosol in both apparel removal rooms that are smaller than <2.5 μm (Figure 1) should remain viable for 3 h with a half‐life of 1.2 h.^59^ However, SARS‐CoV‐2 in the aerosol fraction >2.5 μm in the medical staff office and apparel removal zone C should have remained viable for a longer time (e.g., on plastic and stainless steel surfaces it remains viable for up to 72 h).^59^ The previous information should guide future viability studies for SARS‐CoV‐2 in aerosols of different size, as the information in Figure 1 did not distinguish the percentage of viable virus, which additionally can vary with the environmental conditions and local turbulence and ventilation. Additionally, it will be important to assess how low the concentration of virus, for different particle sizes, can result in infection. For example, long‐term stability experiments of aerosolized SARS‐CoV‐2 (1–3 µm particles at 23°C and 53% RH) have shown the airborne virus remains infectious after 0.17, 0.5, 2, 4 and 16 h.^60^ Scanning electron microscopy studies of collected aerosol samples revealed the virion integrity of SARS‐CoV‐2 (ovoid or spherical morphologies, size and aspect ratio) can be maintained up to 16 h suspended in air.^60^ When considering airborne transmission of the virus outdoors, for the typical volume of air involved in respiration (0.5–1.5 m^3^ h^−1^), the probability of inhalation of viable SARS‐CoV‐2 is very low.^61^

A significant factor believed to enhance the infection caused by SARS‐CoV‐2 seems to be connected to existing underlying diseases (e.g., influenza, cystic fibrosis, smoking, diabetes, etc.), which reduce the rate of clearance.^50^ ^,^ ^51^ ^,^ ^53^ ^,^ ^62^ In other words, other diseases may enhance the residence time for deposited SARS‐CoV‐2 RNA within the respiratory tract. When considering the bioaerosol particles reaching the not‐ciliated tissue covering the lungs, all clearance work is executed by available alveolar macrophages. These macrophages react to phagocytose the particles and transport them to resident lymph nodes, contributing to the fight of the immune system against the virus.^63^, ^64^, ^65^

## 4 FROM A KNOWLEDGE GAP TO PREVENTIVE MEASURES

Understanding the sources, transport, clearance, transmission and inactivation of SARS‐CoV‐2 RNA from emitted bioaerosols is an urgent matter for society. Such effort requires interdisciplinary collaborations, the use of modern techniques, and the implementation of new technological^66^ solutions. There are still limitations in translating the concentration of airborne infectious particles with SARS‐CoV‐2 RNA and their particle size from a single study (Figure 1), to a practical evaluation of infection rates under variable airflow conditions and exposure intervals among other variables. Such valuable information would contribute to reducing COVID‐19 airborne transmission. The general recommendation to keep at least 2 m separation^67^ ^,^ ^68^ from any person showing (or not) symptoms of COVID‐19 (e.g., coughing) should be valid for well‐ventilated environments but fluctuates with the time scale of exposure among other parameters. Fine and ultrafine aerosols that remain in suspension for hours and travel long distances may still transmit a payload of SARS‐CoV‐2 RNA if directly inhaled.

There is a large scientific agreement that COVID‐19 can be transmitted by airborne route effectively. Based on the 3 h viability of SARS‐CoV‐2 in air,^59^ exposure, inhalation and infection could occur minutes or a few hours later near and far from an aerosol source^69^ in a stagnant atmosphere. While the ddPCR method reported the concentration of target sequences along with their Poisson‐based 95% confidence intervals,^18^ further work will be needed to discriminate how much of the detected SARS‐CoV‐2 RNA copies (for each aerodynamic size range) are indicative of viable (transmissible) virus. The high *R*_*0*_ value of the COVID‐19 pandemic, together with the severity of respiratory distress syndrome has severely affected the capacity of hospitals, the operation of office building, the aircraft transportation sector, touristic cruise ships, and hotels among many other similar examples that could be provided. Government officials, advised by responsible health professionals, aim to prevent further population infection by controlling the CODIV‐19 pandemic through the principle of keeping social distancing and quarantine. Preventive COVID‐19 spreading requires using masks in public, sanitization of high‐risk areas and protective medical equipment before removal, and ensuring effective ventilation of indoor environments.^18^ Moreover, the detection of RNA from a series of proxy viruses in exhaled breath and coughs of patients with acute respiratory illness was significantly reduced by face masks for influenza in respiratory droplets and coronavirus in aerosols.^70^ In addition, as there is a large uncertainty in the application of exciting technologies to solve the transmission through bioaerosols, efficient filtration (i.e., with N95 filtering respirators)^71^ will remain the most widely protective equipment used by first health workers.

## 5 CONCLUSIONS

In conclusion, this study indicates the importance of keeping frequent sanitization of high‐risk areas and protective medical equipment, washing hands, wearing masks in public and keeping social distancing to prevent the fast dissemination of SARS‐CoV‐2. While outdoors activities are considered safer than indoors from naturally occurring pathogen dilution in air, and the sanitizing action of sunlight, keeping at least 2 m separation from any person is an effective preventive measure. Such a distance is appropriate for environments that are well‐ventilated but fluctuates with other parameters such as the time scale of exposure. Indoors increase the likelihood for directly inhaling suspended fine and ultrafine bioaerosols with the pathogen, which may still transmit SARS‐CoV‐2 a few hours later. Thus, special consideration is needed for ensuring effective ventilation and filtration of indoors air, disinfection of surfaces and the use of personal N95 respirators by health care workers to avoid the deposition of pathogenic bioaerosol in the respiratory tract.

## CONFLICT OF INTEREST

The author declares no conflict of interest. The funder had no role in the design of the study, or interpretation of data; in the writing of the manuscript, or in the decision to publish this material.

## ETHICAL STATEMENT

This review does not consist of human subject research; no further ethical considerations are reported.

6

## Data Availability

Data sharing is not applicable to this article as no new data were created or analyzed in this study.
